# Matrix transfer techniques for direct paste composite resins

**DOI:** 10.1038/s41415-022-4447-8

**Published:** 2022-07-22

**Authors:** Oliver Bailey, Colin McGuirk, Christopher O´Connor

**Affiliations:** 41415123874001 Clinical Fellow. School of Dental Sciences, Newcastle University, UK; 41415123874002 Private Practitioner, Circus Dental Practice, UK; 41415123874003 Chief Executive Officer, Incidental Ltd, UK

## Abstract

This article discusses potential concerns and important considerations when selecting and using matrix transfer techniques for the direct, definitive addition of composite resin to teeth. It also provides guidance to aid these processes with the use of case studies, primarily focusing on the management of tooth wear.

## Key points

- Considers the advantages and disadvantages of using different matrix transfer techniques for the restoration of multiple teeth with direct paste composite.
- Addresses common concerns and problems associated with using matrix transfer techniques, particularly in relation to managing the worn dentition.
- Suggests guidance on selecting appropriate matrix transfer techniques to minimise complications and optimise outcomes through the exploration of case studies.

## Introduction

Minimally invasive approaches to the restorative management of the worn dentition and aesthetic shape concerns are now widely advocated.^1^^,^^2^ Composite resins are commonly used for these purposes, with various formulations and techniques described, ranging from the direct placement of flowable or paste materials to the placement of indirect restorations.^3^^,^^4^^,^^5^^,^^6^^,^^7^^,^^8^ Each has its place, but this article will focus on the use of direct paste composite, which is perhaps the most evidence-based and adaptable of these materials.^9^

Matrix transfer techniques (MTTs) are commonly used to directly convey planned information from a diagnostic wax-up (either analogue, digital or intra-oral mock-up) to a definitive restoration. The effective use of a MTT will improve the accuracy of the transfer, allowing predictable and efficient treatment. They are used to assist direct placement of paste composite resin to replace lost tooth structure associated with tooth wear or make aesthetic improvements in a conservative and affordable way.

Many different techniques have been described.^3^^,^^4^^,^^6^^,^^10^^,^^11^^,^^12^ While there is no doubt that MTTs offer more control in transferring planned changes to teeth than using a freehand approach, there are varying nuances, advantages and disadvantages relevant to the many techniques. As such, there is no single panacea and different techniques may be suitable for different clinical situations.

These techniques are commonly used to treat multiple teeth simultaneously to make treatment more efficient but they can also be used to aid restoration of single teeth. This article will focus on the simultaneous restoration of multiple teeth, as this is where problems more commonly arise. A highly accurate working impression is important to minimise errors as the working model obtained provides the basis for the transfer. Spaced two-stage (putty/light-body) silicone impressions and optical impressions can provide this. Errors can occur at many stages of the transfer and they can compound, ultimately leading to loss of control and unwanted outcomes, which can be very difficult to manage. Care and precision at each stage is critical to obtain good results.

## Concerns

Common problems associated with using MTTs include:

- Sticking teeth together
- Issues developing anatomically correct, smooth interproximal contacts
- The potential for matrix distortion
- Problems seating the matrix
- Under- or over-filling the matrix
- Managing excess composite
- Achieving isolation of the teeth while allowing seating of the matrix
- Traumatising the gingivae resulting in bleeding impairing resin bonding
- Appropriate staging of transfers
- Achieving stable tooth-borne stops for the matrix or matrices
- Managing existing restorations
- Crowded, overlapped teeth.

Potential MTT options will be discussed and suggested ways to manage some of the concerns will be shown in various case studies.

## Transfer options

MTTs can be used to transfer either part of the planned information only (partial contour) or the full contour. The indications and options for each will be discussed in turn.

## Partial contour MTTs

**Fig. 1:**
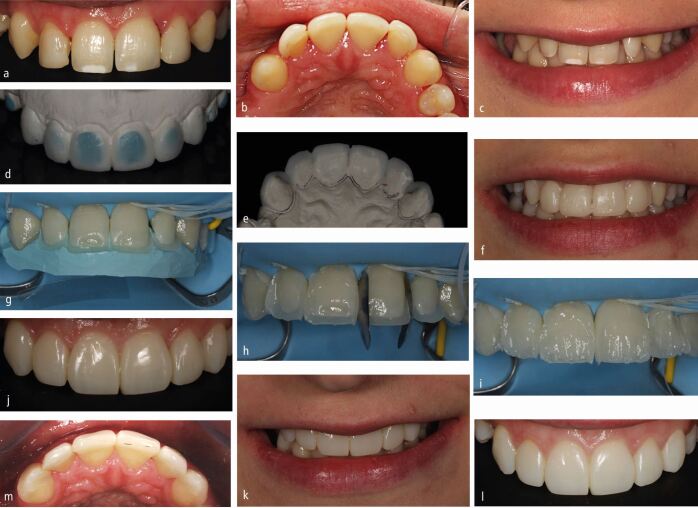
a, b) Previous freehand addition of composite. Patient complained of chipping, gaps, shape issues, centre-line cant, white marks, inability to clean and bleeding gums because of ledges. c) Removal of existing composite. d, e) Wax-up to address these requests following removal of previous composite additions. f) Mock-up to assess aesthetics, functional occlusion and speech using temporary crown material in a putty of the wax-up. Patient happier with this but midline cant still evident. Patient informed of possible incomplete resolution without preparation, which was accepted. g) Proposed additions light-curable from buccal, aesthetic shade layering planned, therefore rigid opaque matrix used for partial transfer made from bite registration silicone. Partial 'shell' transfers, allowing aesthetic shade layering. h) Centre-line cant correction and control over gingival contour through use of contoured interproximal metal matrices. i) Layered approach. j, k) Post-operative result. l, m) Two-years follow-up, showing improved gingival health and acceptable maintenance of aesthetics before re-polishing

Partial contour MTTs commonly transfer just the palatal or lingual shape of the planned information (the 'shell') (Fig. 1e). The shell is particularly useful for upper anterior teeth as this defines the planned functional surfaces in addition to facilitating aesthetic composite layering techniques. This technique also allows for separate formation of smooth anatomical contact points. The ability to control each aspect of the build-up has the advantages listed above; however, the compromise is that this technique will tend to be more time-consuming.

### Aesthetic shade layering

Partial transfers are advised when composite resin aesthetic shade layering is required. This allows control over layer thickness of the different composite shades and opacities, which allows for optimal aesthetic outcome and may be critical to the success of such restorations (Fig. 1).^11^

### Formation of contacts

**Fig. 2:**
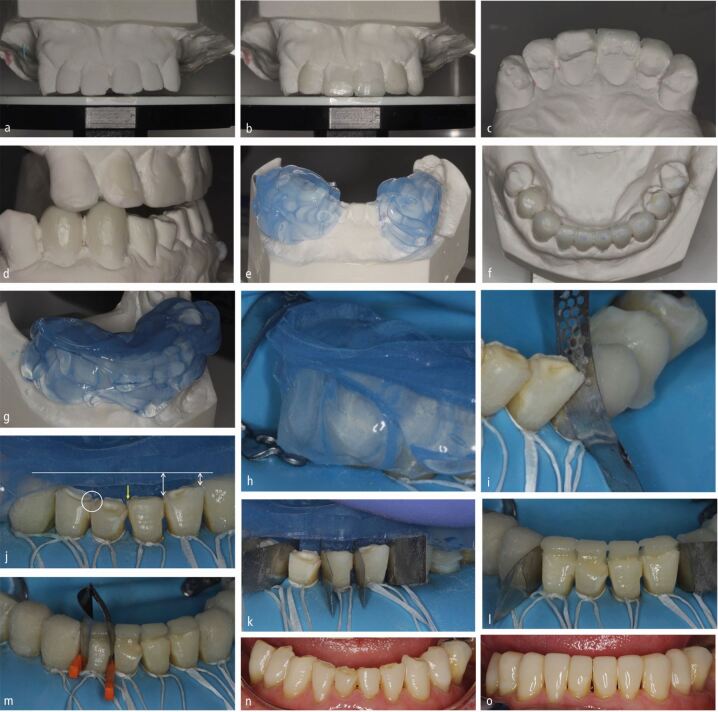
a, b, c) Wax added buccally only to determine proposed final shape of maxillary anteriors. d) This allowed the required opening of vertical dimension to be planned with the casts mounted in centric relation on a semi-adjustable articulator. This was then stabilised with additions to the upper canine and lower first premolar and canine teeth bilaterally. e) Matrices were then made before completing the wax-up, allowing these changes to be transferred first. This allowed unrestored teeth to be used to stabilise these initial matrices at the first appointment. Full contour MTTs were planned for the posterior teeth, as the contact points were intact. f) The wax-ups were completed. g) New matrices made (lower arch process shown as an example). h) Posterior unvented flexible silicone full contour matrices stabilised on unrestored teeth. i) Excess directed unfavourably - cervically and towards embrasures, requiring separation using metal interproximal strips. j) The lower anterior teeth were crowded (yellow arrow) and had suffered differential wear, (white line and white arrows) making the seating of full contour MTTs difficult if wishing to incorporate metal separators which would require a POI from the incisal (yellow arrow). Preparation of the teeth to remove the crowding (yellow arrow) and open the embrasures (white ring) to allow a mutual incisal POI could have allowed the use of a full MTT with metal separators with mutual incisal POIs. k) A partial MTT was therefore chosen, which allowed the use of metal separators, because of the less constrained POI of the matrix from the lingual aspect. l, m) Lingual shells were built, allowing the use of contoured metal matrices and wooden wedges to separate and build the interproximal aspects of the teeth, favouring the formation of smooth contacts. If using a combination of full and partial MTTs, it is better to complete the full contour restorations first, allowing the partial transfer matrices to be stabilised on the more accurate full contour additions. n, o) Before and after

**Fig. 3:**
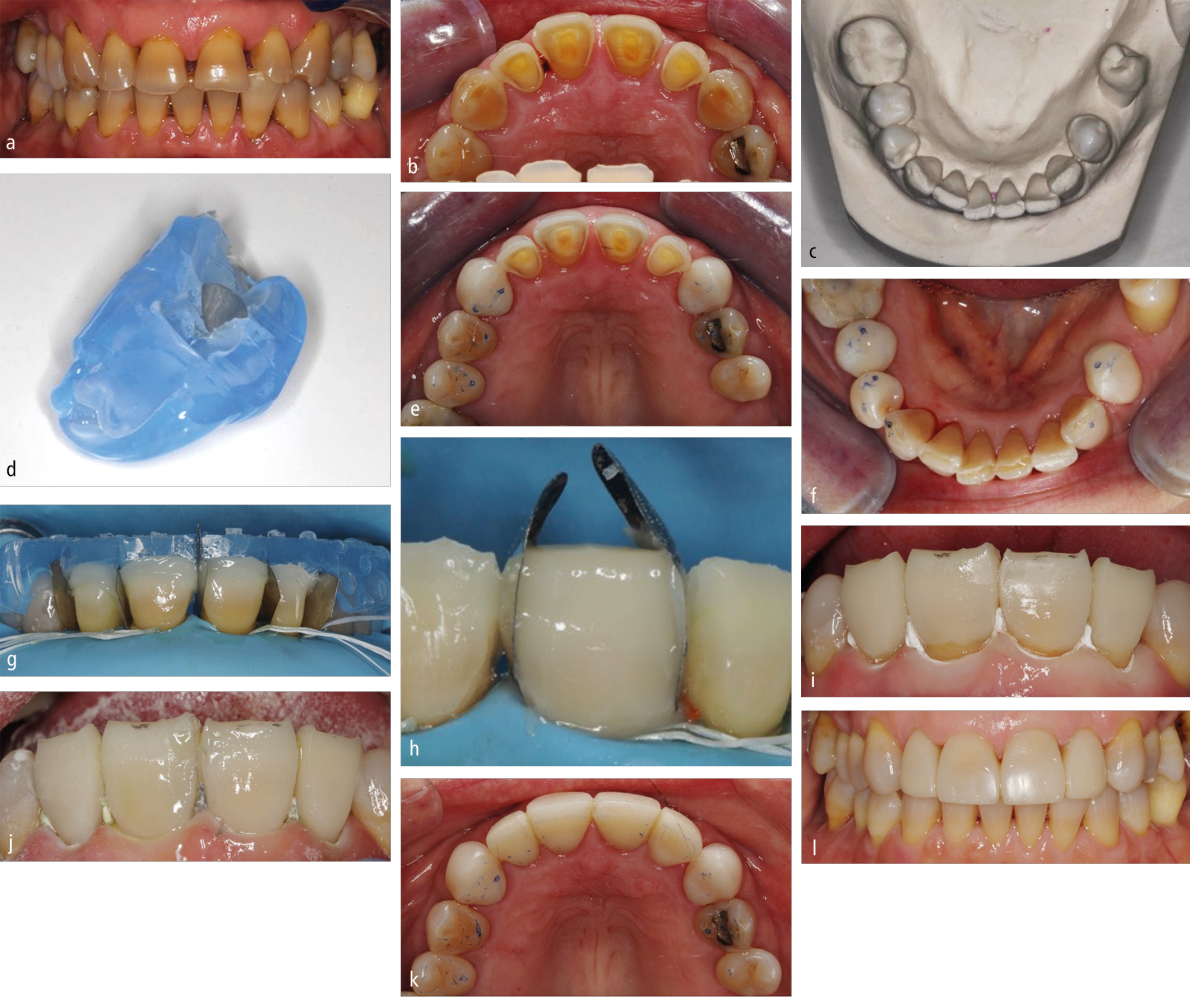
a, b) Contacts not intact anteriorly. Patient desire to close diastemata. Partial shell MTT planned to allow development of contacts. Palatal composite not accessible for curing from buccal, therefore clear matrix required. c) Contacts intact posteriorly. Wax additions just shy of contacts. d) Clear silicone matrix unvented with metal separators added to prevent sticking teeth together (same for upper canines following aesthetic edge wax-up to determine new OVD). e, f) Premolar and canine teeth restored first appointment with full transfers. g) Maxillary incisors restored using clear silicone partial MTT with metal separators to prevent sticking teeth together and to ensure planned space for each tooth not impinged upon. Matrix stabilised on previously restored canines placed with full contour MTT. Heated composite placed and cured from palatal. h) Contact areas developed with contoured metal matrices in combination with wooden wedges for separation and cervical seal. i, j) Interproximal and facial areas overbuilt due to freehand composite addition, which would not allow accurate subsequent seating of a matrix if these were completed first without taking new records, hence the initial placement of the canine additions using a full contour MTT. Rubber dam isolation hadn't fully isolated the clinical crowns. The cervical regions were therefore subsequently restored following isolation and retraction with PTFE tape. k) Post-operative outcome. l) Two-year follow-up

Partial contour techniques may also be better employed when there is no existing contact between the teeth to be restored, such as in diastema closure cases. Solely transferring a shell allows the interproximal contours to be built with anatomically shaped matrices, which are easily stabilised in an appropriate position because of the proximity of the shell to the adjacent tooth (Figures 1f, 2j and 3f). These matrices can also be sealed cervically and adjacent teeth sufficiently separated with a wooden wedge to obtain a contact (Figures 2j and 3f).^13^^,^^14^ This allows the predictable formation of contact areas when the interproximal matrix is removed and produces smooth, cleansable interproximal emergence profiles with minimal excess, which reduces restoration finishing time (Figures 1f, 1g, 1h, 2j, 2k, 3f, 3g, 3h, 3i).

### Partial contour matrix material choices

#### Access for light curing

The matrix material to be used for partial transfers is dependent on whether the palatal composite to be added is accessible from the buccal aspect to allow light-curing. If it is, an opaque matrix can be used. Putty is often used but the authors favour bite registration silicone, as it is more rigid and therefore less prone to distortion (Fig. 1e).

**Fig. 4:**
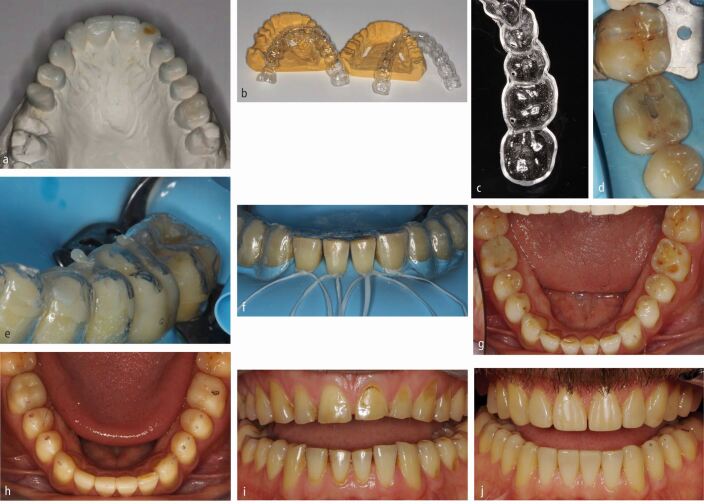
a) Wax additions not extended to interproximal regions posteriorly, matrix stabilised on uncovered interproximal tooth tissue - full transfer used. Wax extended to contact anteriorly - partial transfer used. b) Wax-ups duplicated in stone, to allow fabrication of rigid plastic matrices. c) Surface detail comparatively poor compared with silicone materials. Vented distant from occlusal contact with same trajectories to facilitate removal of matrix. d) Retention grooves cut into intact existing restoration 46 (and surface sandblasted). e) Full transfer with heated composite, rigid vented plastic matrix and heated composite. f) Full contour posterior additions placed first allowing accurate seating of matrix for anterior partial transfers. Anterior spacing to be closed, therefore partial lingual transfer as previously shown. g, h, i, j) Post-operative outcome

**Fig. 5:**
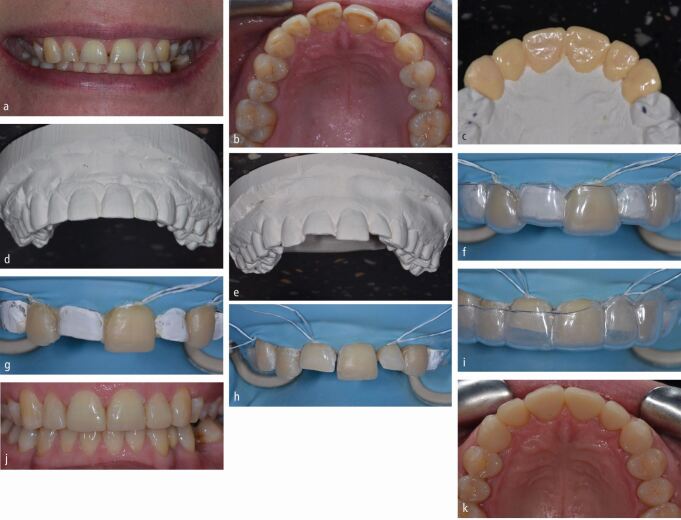
a, b) Alternate tooth full contour MTT. Clear matrices required re full contour. c) Wax-up of all teeth performed. d) Wax-up duplicated in stone. e) Wax removed from every other tooth and duplicated in stone. f) Rigid unvented matrix with every other tooth waxed up used first to allow stable seating stops on unrestored teeth. PTFE tape placed to prevent bonding composite to adjacent teeth. g) Immediately after full contour placement of alternate teeth with heated composite. h) Finishing completed interproximally. i) Second matrix made on full wax-up subsequently used, stabilised on restored teeth. j, k) Post-operative outcome

**Fig. 6:**
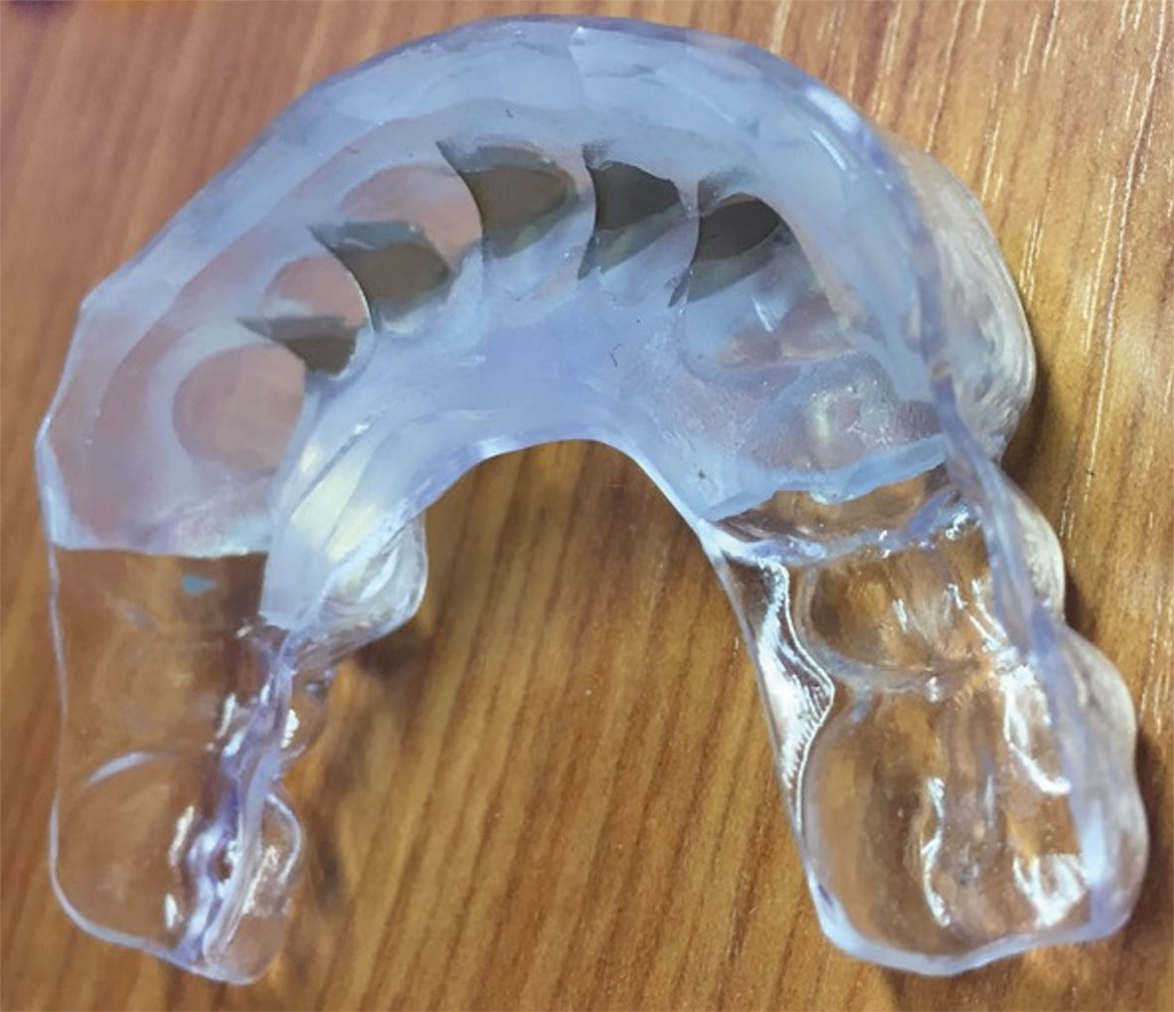
Combined silicone and thermoformed plastic clear matrix with metal separators

If the palatal composite is inaccessible from the buccal to light-curing, a clear matrix is required to allow the composite to be light-cured from the palatal (Figures 3a and 3e). Clear silicones can be used, though they often display increased flexibility and are therefore prone to distortion on seating or when stabilising the matrix. This means that the shape of the restoration placed may not be as planned and excess composite may be displaced towards the contact area or cervically (Figures 2f and 2g). They should therefore be used in thick section or can be placed in a pressure pot to improve their rigidity. Thermoformed plastic shells can be used alone (Figures 4b and 5e) and though they are extremely rigid, thus minimising the potential for distortion, they can lack in surface detail compared to the silicone materials and can be difficult to remove. The benefits of each material can be exploited by using them in combination, where an inner layer of clear silicone is encompassed by a clear rigid plastic outer shell (Fig. 6).^3^

When substantially augmenting the palatal aspect of teeth, it is important to consider and appropriately plan the matrix stops, which should be tooth-borne to allow accurate seating of the matrix. This planning is important because the remaining tooth tissue may need to be completely overbuilt and cannot therefore be used to stabilise the matrix (Fig. 2a). If this is not taken into consideration, the matrix will be supported on soft tissue, which is potentially compressible. This, in turn, leads to uncertainty as to whether the matrix is seated, which could result in over- or under-seating and associated shortened or elongated composite additions. It is also important to consider the staging of transfer of restorations (that is, the order in which the restorations will be placed), which can allow treatment to occur over multiple visits if required.

Staging of the transfer commonly requires consideration when using full contour MTTs (see later) and especially analogue wax-ups (that is, when wax is added to a stone model) (Figures 2a, 2b, 2c, 2d, 2e). When following a facially generated treatment planning approach, whereby the treatment approach is dictated by the planned shape and position of the anterior teeth in relation to the lips and face,^15^ an aesthetic wax-up can be performed initially to aid planning.^4^ This involves wax additions to the buccal aspect of the upper anterior teeth only, without yet waxing up the palatal aspect (Fig. 2a). The required opening of occlusal vertical dimension (OVD) to functionally accommodate these planned aesthetic changes in the teeth can then be determined (Fig. 2b). The posterior teeth can then be fully waxed at this new OVD (as required), leaving unrestored anterior tooth tissue for use as matrix stops (Fig. 2a). A matrix can then be fabricated involving these, as yet, unwaxed surfaces (Fig. 2c). The wax-up is then continued (in the order of the planned stages), making new matrices for each section, eventually using the restored teeth as stops to transfer the final additions (Figures 2e and 3e).

Digital wax-ups allow this planning of staging to take place after the wax-up is complete, as parts can easily be digitally subtracted and multiple segmented matrices made. Another advantage of the digital technique is that modifications to the wax-up can easily be made after it is finished, without potentially ruining previous matrices (as can occur with the analogue method). It often only becomes apparent that modifications are required when the wax-up is mocked-up in the patient's mouth. An intra-oral mock-up is produced by making a full contour matrix of the wax-up which is then used to reversibly transfer temporary crown and bridge material to the patient's teeth, which allows the planned outcome to be critically appraised by patient and operator (Fig. 1d). Any modifications required can therefore easily be made before fabricating the definitive matrices when doing this digitally.

## Full contour MTTs

Full contour MTTs allow placement of all of the planned shape in composite at once. These matrices therefore must allow light transmission to facilitate light curing with the matrix in place. Single shade restorations are more commonly placed with full contour MTTs, as layer thickness is difficult to control.

Full contour MTTs can potentially improve efficiency, especially when multiple restorations are placed together, but do have a tendency to create more excess with an increased potential for sticking teeth together, making the finishing more difficult and time-consuming. Staging of the transfers, with a consideration of matrix stops, is also often required, as previously discussed. Techniques have therefore been developed in an attempt to overcome these issues. The methods can be described as:

- All at once shy of the contacts
- All at once with metal separators
- The alternate tooth technique.

### All at once shy of the contacts

It is often prudent, where possible, to wax planned additions just shy of the contact (Figures 3b and 4a).^3^ Obviously, this requires that the contacts are intact, or that spacing is to be accepted. This gives stable tooth-borne stops to effectively seat the matrix and aims to avoid sticking adjacent teeth together. Using a matrix with high rigidity is a critical element when relying on this technique to avoid this complication, as loading of the matrix with excess composite (which is necessary to prevent voids in the restoration), or distortion of the matrix on seating, will potentially allow excess composite to flow to adjacent teeth. Minimising the seating pressure as much as possible is also important to reduce matrix distortion and allow full seating. Use of heated composite, which improves the flow characteristics of the material, will aid this, as will creating vent holes in the matrix to allow extrusion of excess composite (Figures 4c, 4d and 4e). Venting is best placed occlusally or incisally away from the planned occlusal contact so that when the excess from the vent hole is removed, the planned occlusal contact isn't inadvertently adjusted. The trajectory of the vent holes should also be aligned on all teeth when multiple restorations are to be placed at once, facilitating removal of the matrix. Failure to vent a matrix allied with a lack of matrix rigidity will tend to push the excess more gingivally and towards embrasures, potentially sticking teeth together, which can be difficult to manage (Fig. 2g).

### All at once with metal separators

This gives the operator confidence that the teeth won't be stuck together (Figures 3c and 6), but the major issues are that it can be difficult to seat such matrices and if contacts don't exist pre-operatively, they will not be created with this technique.

Seating matrices with metal separators can be difficult because of the need for a common path of insertion (POI). This becomes very constraining when restoring the full contour of the tooth, as the matrix must be inserted from the occlusal. It therefore requires aligned teeth; any overlapping will often preclude their use because there will not be a common occlusally or incisally oriented POI (Fig. 2h). Another potential barrier to seating is when adjacent teeth have worn to the contact area, such that there is no open embrasure space coronal to the contact (Fig. 2h). This means that there is no guiding of the metal separator into and therefore through the contact area. There is therefore a tendency for the metal separator to resist placement through the contact and become distorted. This then prevents seating of the matrix. A similar difficulty can occur when there has been differential wear, such that there are occlusal plane discrepancies, as some separators may pass through contacts before others come to a contact (Fig. 2h). This may then make the passage of these separators difficult at the more worn teeth and again tend to distort the separator. Distortion of a metal separator on attempted seating will result in the inability to seat the matrix and necessitates either its removal and the abandoning of the use of that separator, or placing a new undistorted separator chairside and trying again.

Metal separators can be added into a silicone matrix after fabrication by making a slit at the embrasure and then pushing them in (Fig. 3c). Alternatively, they can be incorporated during the fabrication of the matrix on the model (Fig. 6). Grooves need to be prepared between the teeth on a stone duplicate of the waxed-up model, allowing separators to be placed. The separators should have retentive holes, allowing them to be picked up and incorporated within the matrix as it sets.

Preparation of teeth to open up tight embrasures and reduce overlap of crowded teeth can be performed to allow a common POI and therefore eliminate some of these issues. This may be reasonable if the sacrifice of sound tooth tissue and the attendant potential biological costs are deemed acceptable by the operator and the patient; however, inadvertently stripping a contact is a possibility here. If this occurs, the use of a metal separating strip will result in a gap between the teeth when it is removed after placement of the restorations, with the potential for food packing.

This technique works particularly well when it can be combined with the techniques described previously in the 'all at once shy of the contacts' section - planning addition of material just shy of the contacts, using venting and heated composite. It provides an additional safety mechanism to prevent sticking the teeth together but makes insertion of the matrix more difficult. In general, increasing the number of teeth that are restored together will increase the potential for problems with seating.

Metal inserts should be appropriately gingivally extended and contoured to avoid trauma to the gingivae (Fig. 6), which could cause bleeding and interfere with the bonding process.^16^

While tooth wear often results in loss of the embrasure from the incisal, it is rare to lose the palatal or lingual embrasure completely (Fig. 3a). Partial contour matrices with incorporated metal separators can therefore commonly be seated from the palatal or lingual much more easily than full contour matrices (which have to be inserted from the occlusal or incisal), as the embrasure guides the separator into and through the contact (Figures 2i and 3e). It can be useful to incorporate metal separators into a matrix when employing the partial contour MTT, especially when building bulky palatal shells, to prevent the composite flowing onto adjacent teeth when seating a matrix (Fig. 3e). This reduces the risk of sticking adjacent teeth together when seating multiple shells at once and ensures that the space planned for each tooth is not encroached upon. Partial contour techniques may be less time-efficient in such situations, but anecdotally, can result in a more predictable treatment flow and superior outcomes.

### Alternate tooth technique

This involves waxing up all of the planned teeth, duplicating this model in stone and then removing every other wax addition which has been added to the teeth.^6^ This model, with every other tooth waxed, is then duplicated in stone. Clear matrices are then made on both of the duplicated stone models so that there are now stops on sound natural tooth tissue for stable seating of the first matrix (made on the model with every other tooth's wax addition removed) (Fig. 5). Obviously, this is more easily done with a digital wax-up. Each composite addition is then finished while accessible, before covering these restored teeth with polytetrafluoroethylene (PTFE) tape and using the second matrix, which now uses stops on the already restored teeth (Figures 5f, 5g, 5h and 5i). The main advantage of this technique is that the stent will always be tooth supported at regular intervals, even when extensive modification to the supporting occlusal/palatal surfaces are planned. It is also useful for worn teeth which are crowded and overlapping where conventional interproximal matrices are difficult to place without distortion.

Not using an interproximal matrix means that the interproximal contour of the final restorations can be a little rough and prone to staining. While this can be managed with discs and interproximal strips for example (Fig. 5g), there is always the possibility of losing a contact, especially when using strips. Nevertheless, this technique can work well (Fig. 5i).

## Combined techniques

Different MTTs can be used on the same patient, based on the specific situation and staging required. When full contour and partial contour MTTs are to be used on the same patient in the same arch, it is advised to complete the full contour restorations first (Figures 2, 3 and 4). This is because subsequent matrices which have been planned to be stabilised on the composite additions previously placed are likely to seat well on restorations placed with full contour MTTs, as little adjustment is required to the occlusal surfaces after they have been placed. In contrast, where a shell is placed, the facial and interproximal areas are subsequently restored freehand and these are inevitably overbuilt and require finishing back (Fig. 3g). They will therefore differ from the wax-up and such restorations cannot therefore be relied upon as stops for the use of subsequent matrices.

## Bonding considerations

Rubber dam (RD) is usually considered the most effective means of isolation to optimise bonding procedures. Seating of the matrix can be impaired by both the RD and clamps. These issues can usually be overcome with appropriate trimming of the matrix and clamp selection and positioning (Figures 2f and 4e). In some cases, it may be more appropriate to perform all or part of the restorations using appropriate non-RD isolation (Fig. 3g).

Teeth being restored with MTTs often have existing restorations. This raises a dilemma of whether and when to remove and replace them, or accept and add to them. The integrity of such restorations is obviously critical to this decision but also the restoration type, material and future plan for the dentition. If a restoration is deemed intact, it can be added to either as an interim or definitive measure.

Any existing restoration (including indirect restorations) can be managed by cutting retention (Fig. 4d) and implementing an appropriate adhesive strategy.^17^ If easily achievable, it is generally advisable to expose enamel where it is covered by an existing restoration to optimise the adhesive retention of the restoration.^2^^,^^17^^,^^18^^,^^19^^,^^20^ Bonding to dentine is less predictable and predictably breaks down over time.^21^ It should not be relied upon for adhesive retention;^2^^,^^20^ therefore, removing a restoration simply to expose dentine to bond to is likely to be less critical and may therefore be avoided (Fig. 4d).

If the integrity of an existing restoration is doubted, it should be removed and replaced. This can often be performed simultaneously using the selected MTT for anterior teeth and can often be incorporated into a palatal shell. Posteriorly it can be more difficult however. Where a posterior interproximal wall has been lost, it is prudent to restore the contact with an interproximal matrix first,^14^ ensuring the gingival tissue is appropriately managed.^22^ Care should be taken to underfill the cavity in relation to the wax-up. The selected MTT can then subsequently be used but the matrix should be checked to ensure it still seats. If it doesn't, it will be necessary to adjust the restoration until it does.

## Conclusion

There are many different methods for transferring planned information to the mouth using direct paste composite materials, each with various advantages and disadvantages. Partial shell transfers are preferable for anterior teeth where shade layering and/or contact development is required. Using pre-contoured metal interproximal matrices with wooden wedges to build interproximal contours and contacts may minimise the finishing required and produce superior contacts. When using full contour transfer techniques, heated composite with vented matrices and metal separators (where possible) should be considered, alongside waxing shy of the contact. These help to prevent sticking teeth together and favour coronal excess which is easier to manage. When staging of transfers will be required, digital wax-ups are beneficial and full contour transfers should be performed before partial transfers to ensure seating of the matrices.
